# Supplementary material for: Anion-capped metallohost allows extremely slow guest uptake and on-demand acceleration of guest exchange
Source: Nat Commun. 2017 Jul 12;8:16005. doi: 10.1038/ncomms16005 (PMC5510176; doi:10.1038/ncomms16005)
Supplement: Supplementary Information [file ncomms16005-s1.pdf]

Title of file for HTML: Supplementary Information
Description: Supplementary Figures and Supplementary Tables

Title of file for HTML: Peer Review File
Description:

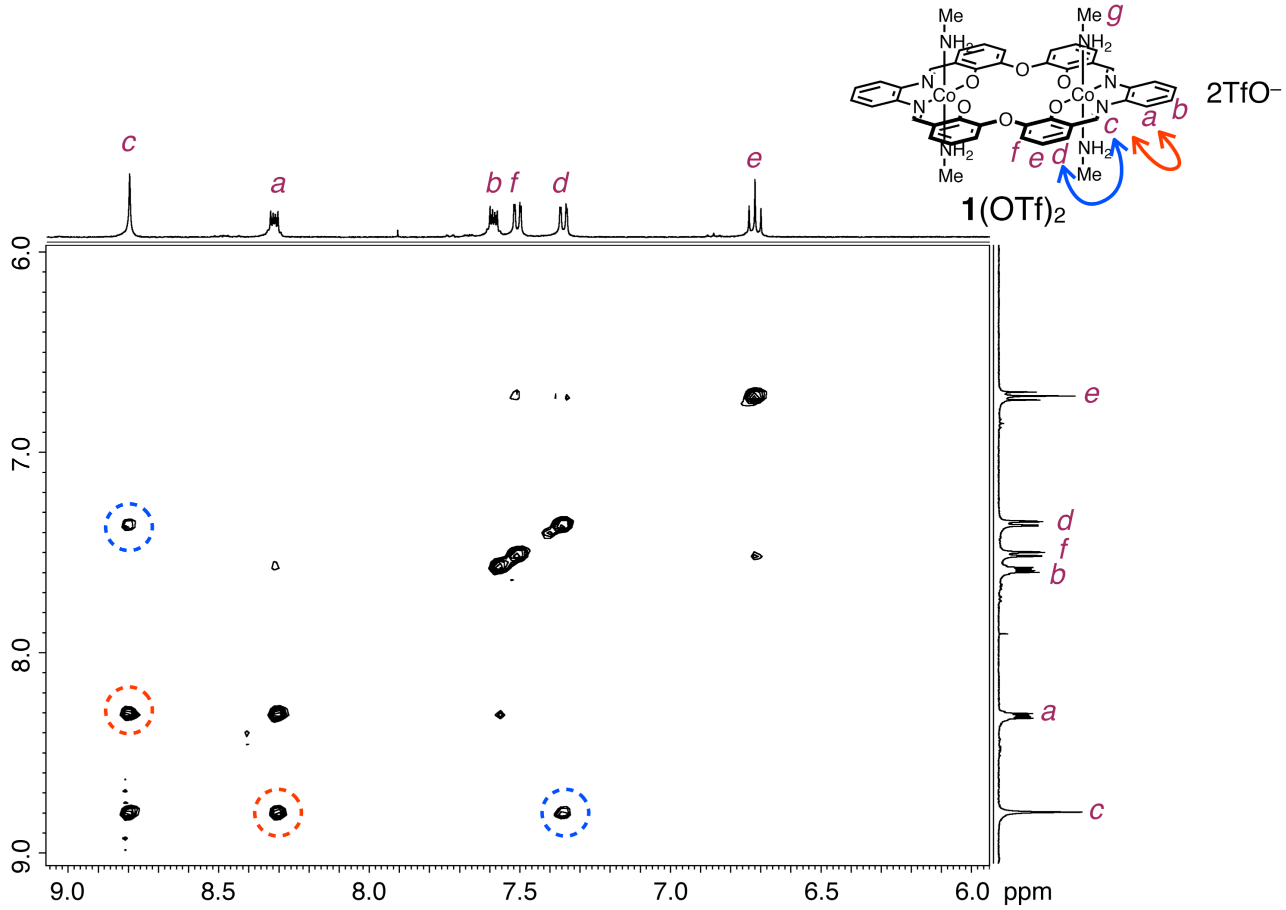

Supplementary Figure 1. NOESY spectrum of **1**$(OTf)_2$.

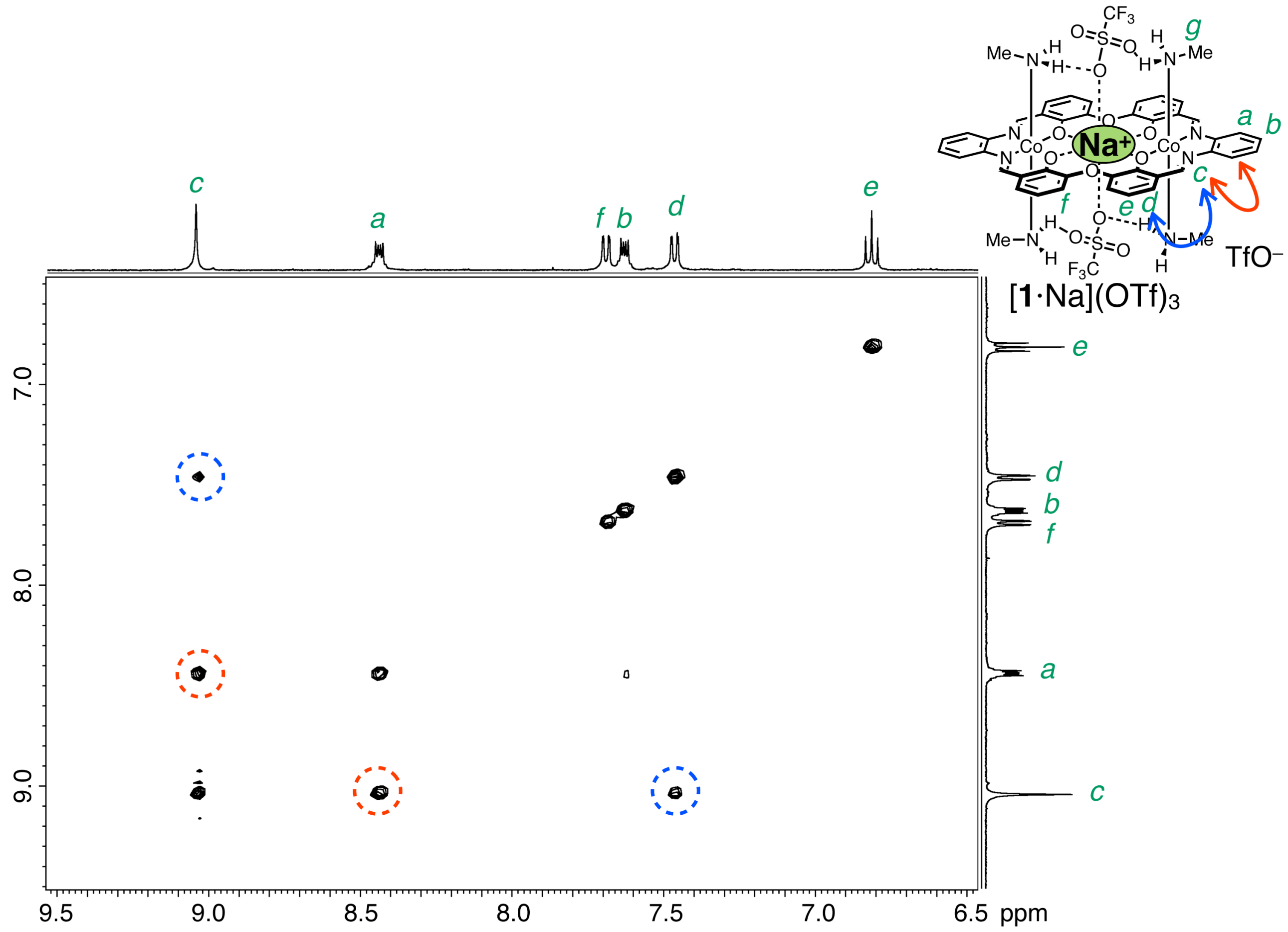

Supplementary Figure 2. NOESY spectrum of [**1**•Na(OTf)$_2$](OTf).

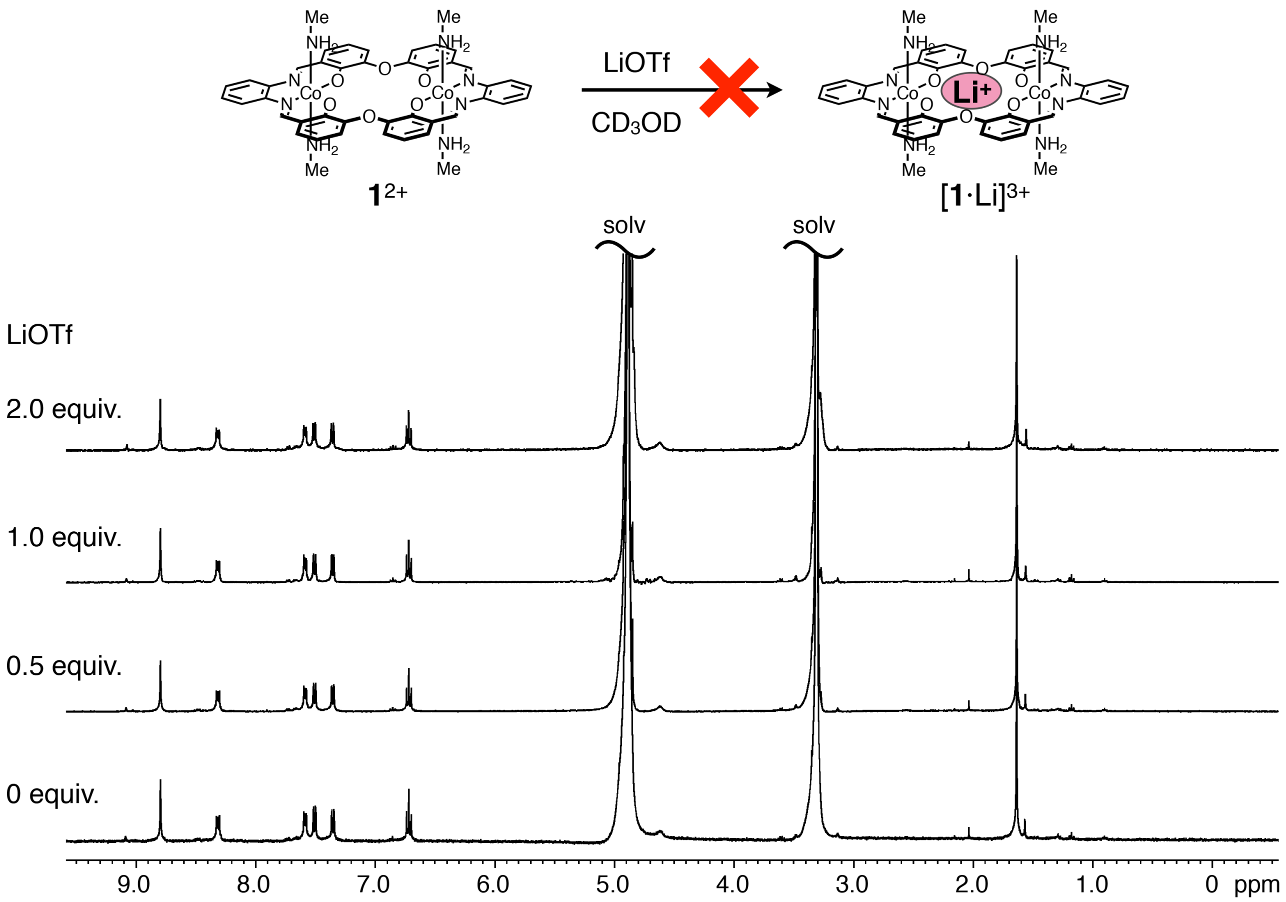

Supplementary Figure 3. ^{1}H NMR spectral changes of **1**$(OTf)_2$ upon the addition of LiOTf (400 MHz, CD_3OD, 1 mM).

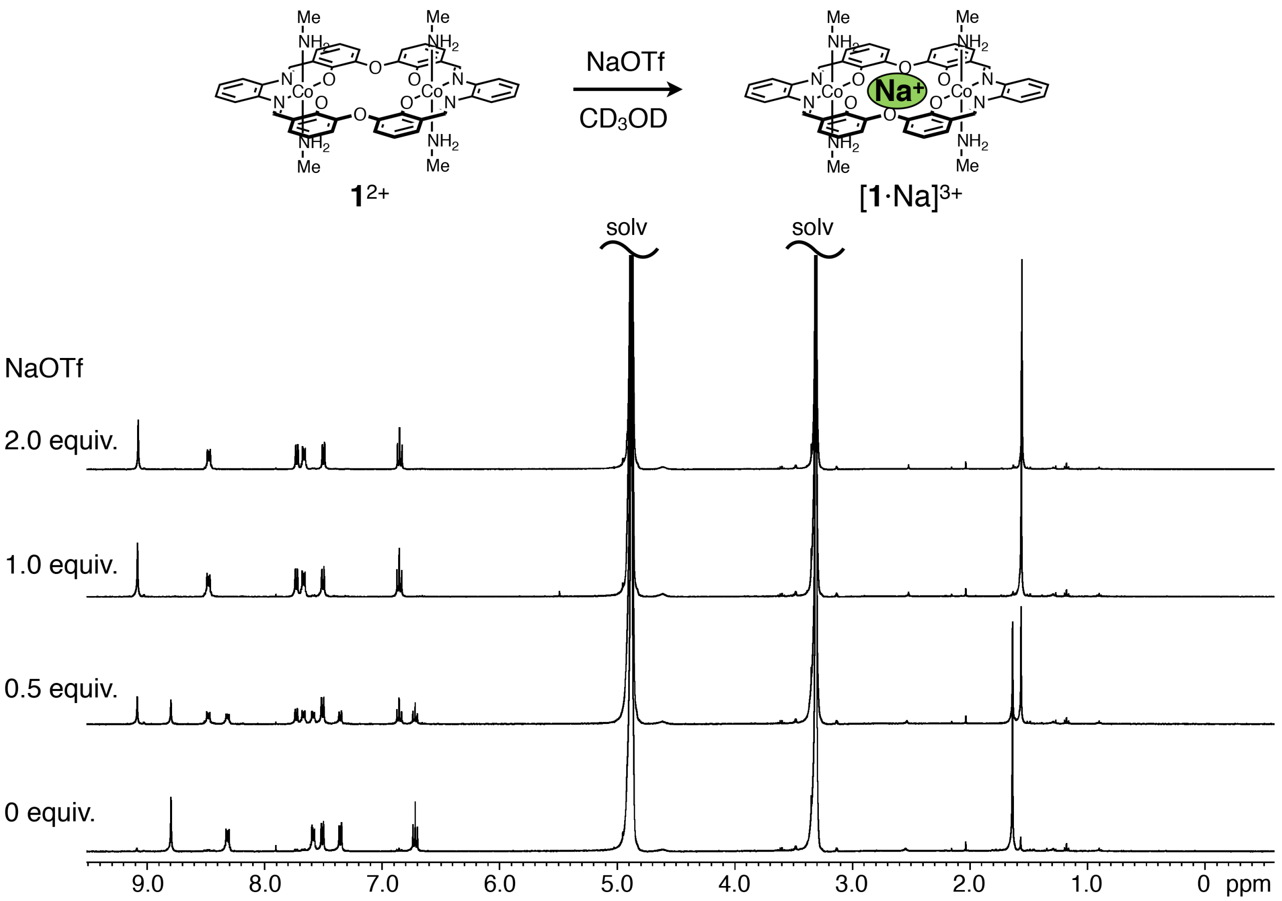

Supplementary Figure 4. ^{1}H NMR spectral changes of **1**(OTf)$_2$ upon the addition of NaOTf (400 MHz, CD_3OD, 1 mM).

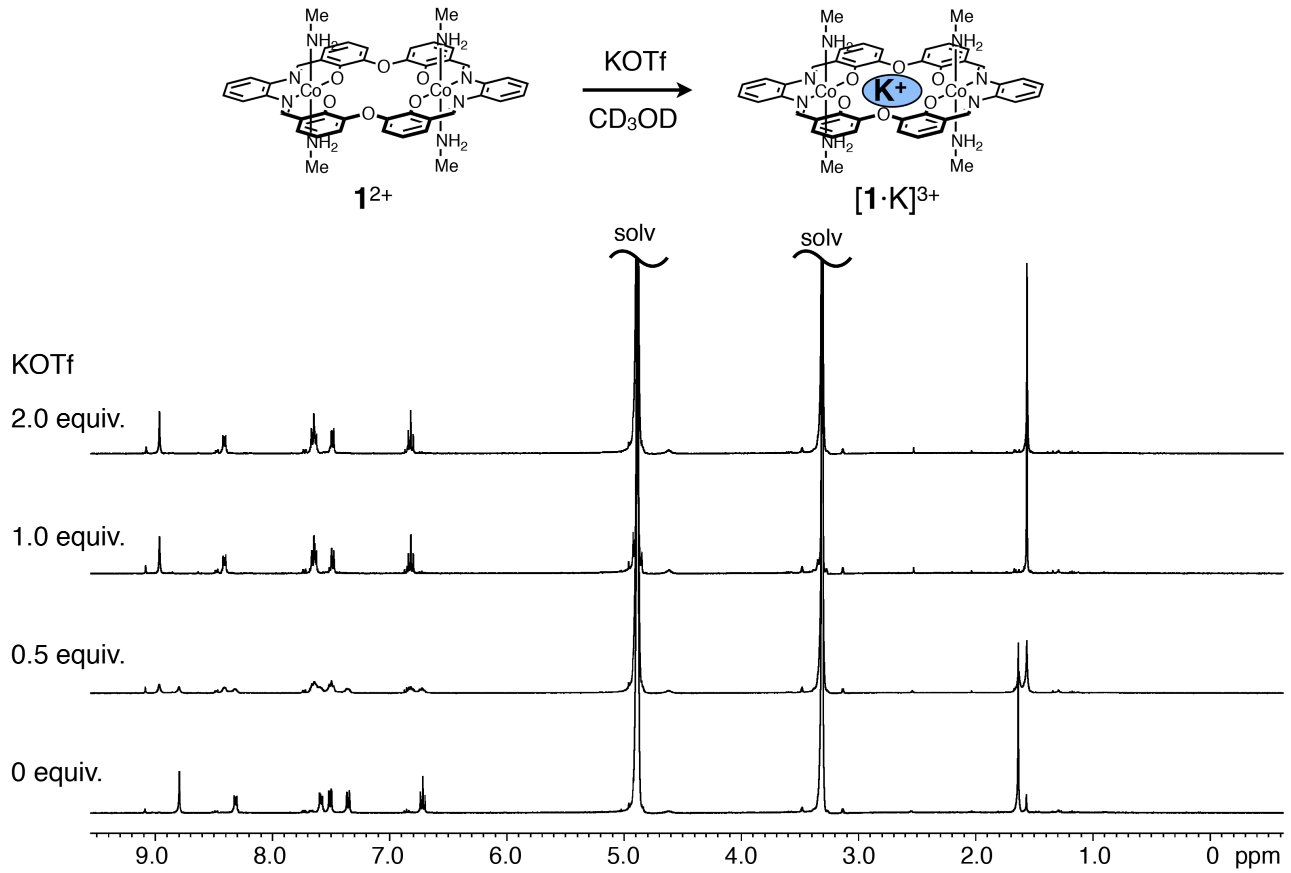

Supplementary Figure 5. ^{1}H NMR spectral changes of **1**(OTf)$_2$ upon the addition of KOTf (400 MHz, CD_3OD, 1 mM).

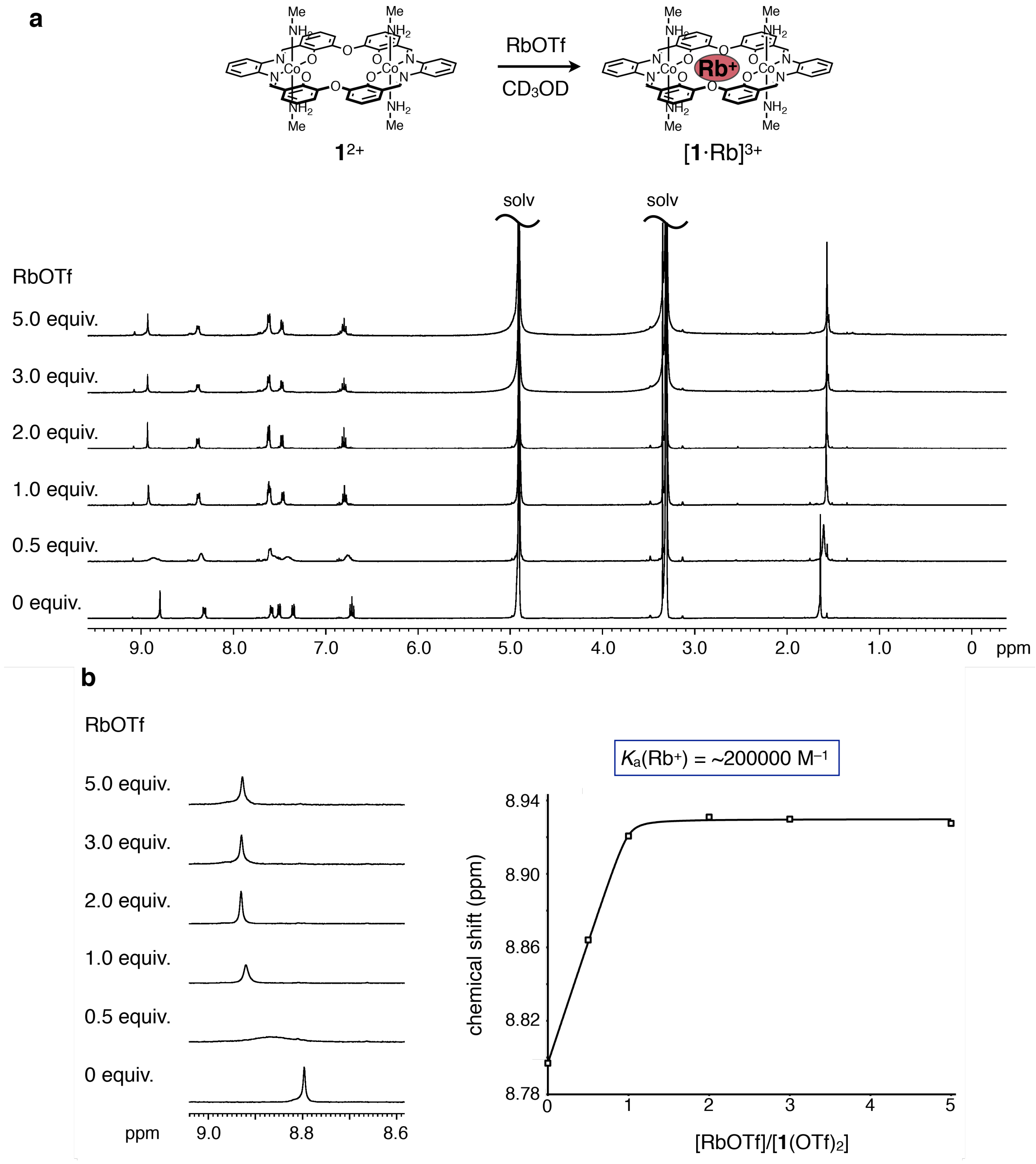

Supplementary Figure 6. a, ^{1}H NMR spectral changes of **1**$(OTf)_2$ upon the addition of RbOTf (400 MHz, CD_3OD, 1 mM). **b**, Nonlinear curve fitting of chemical shifts obtained from the titration study.

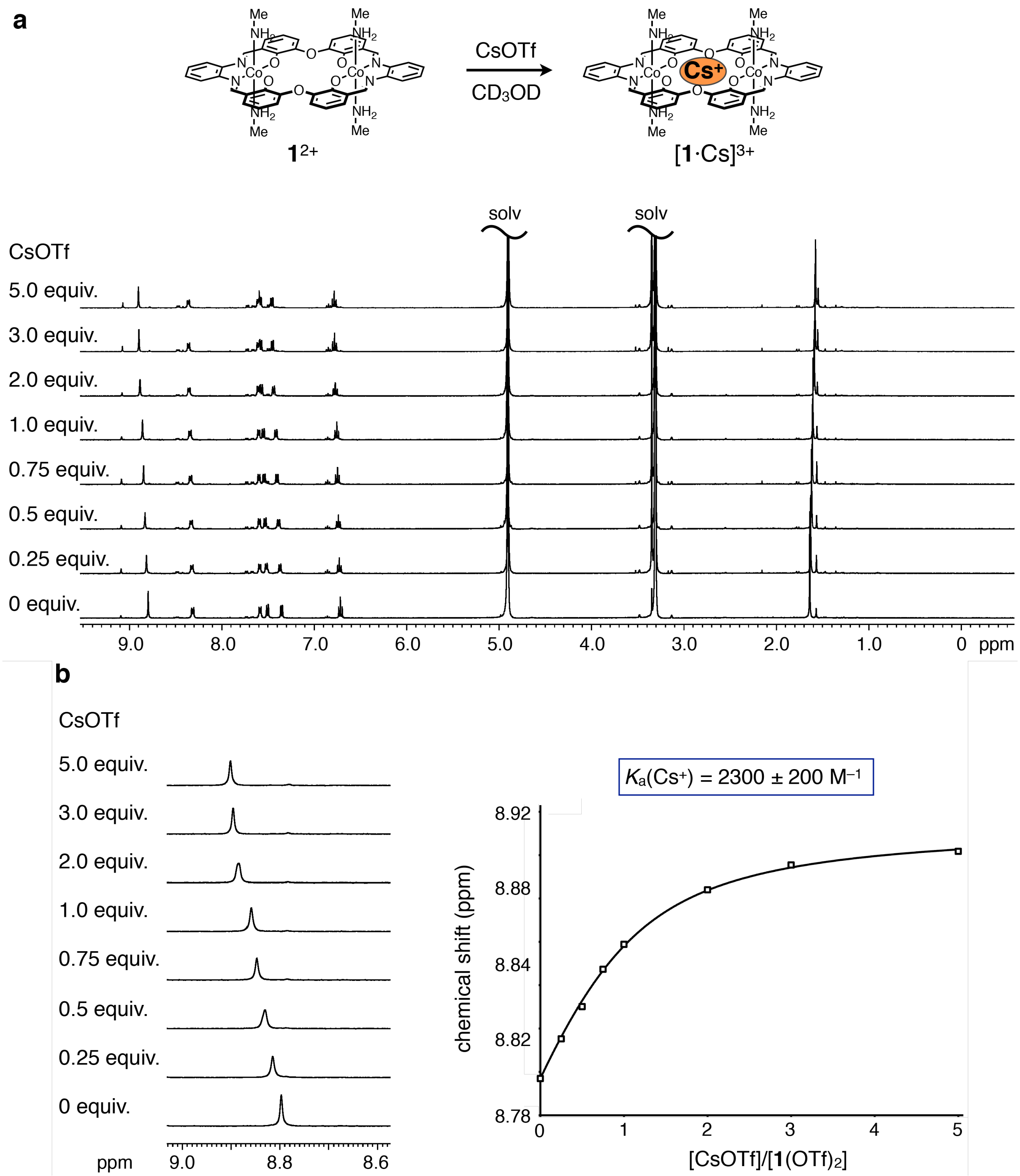

Supplementary Figure 7. **a**, ^{1}H NMR spectral changes of **1**$(OTf)_2$ upon the addition of CsOTf (400 MHz, CD_3OD, 1 mM). **b**, Nonlinear curve fitting of chemical shifts obtained from the titration study.

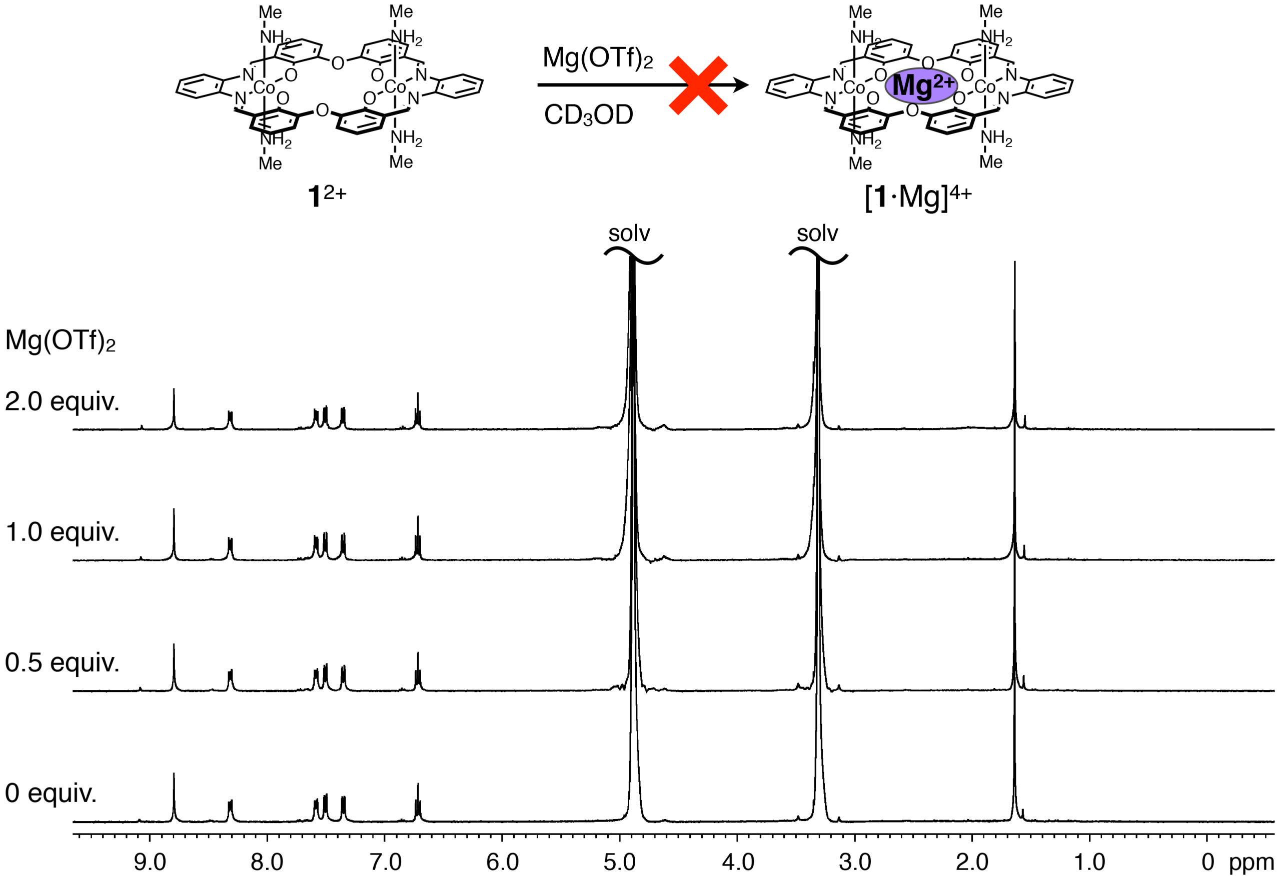

Supplementary Figure 8. ^{1}H NMR spectral changes of **1**(OTf)$_2$ upon the addition of Mg(OTf)$_2$ (400 MHz, CD_3OD, 1 mM).

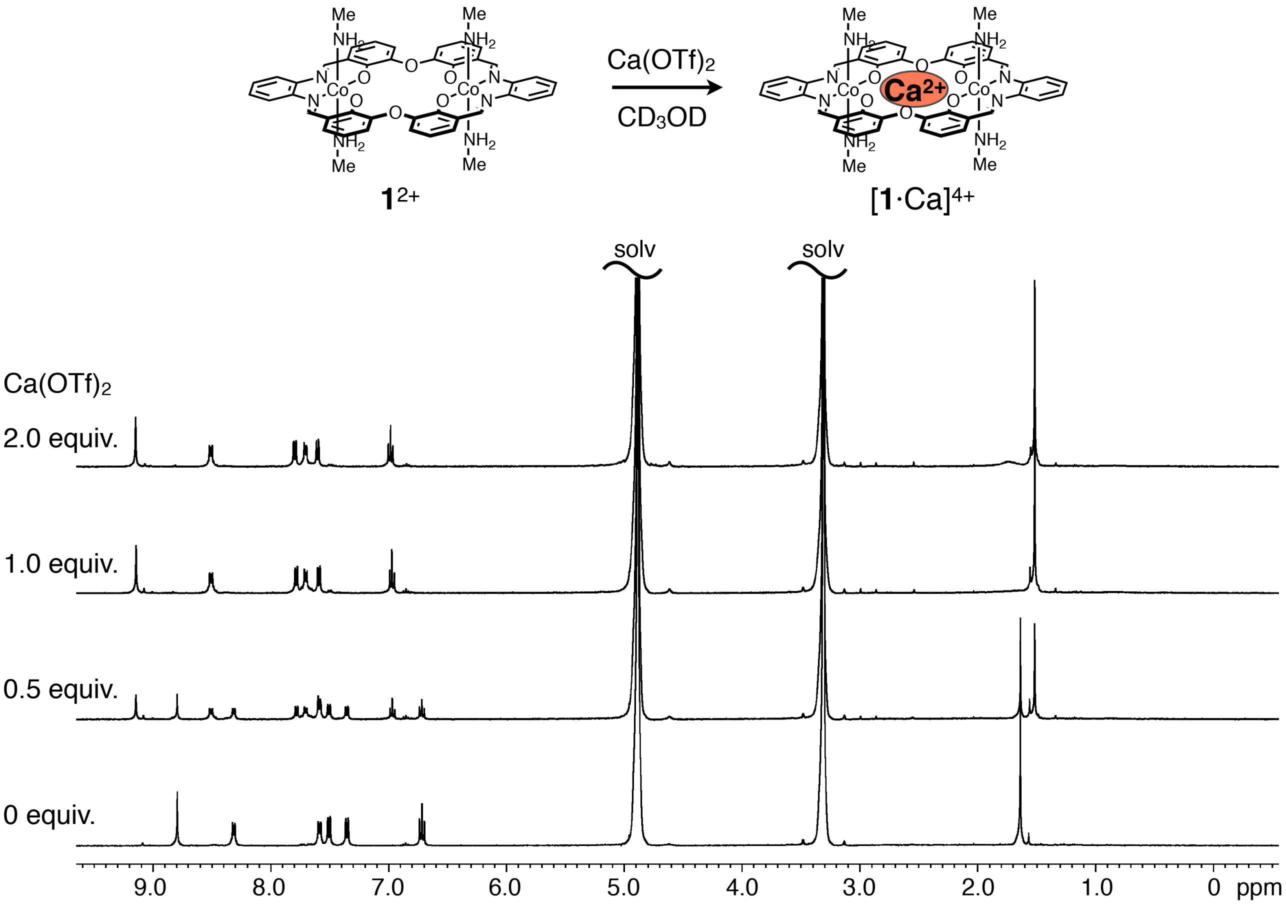

Supplementary Figure 9. ^{1}H NMR spectral changes of **1**$(OTf)_2$ upon the addition of $Ca(OTf)_2$ (400 MHz, CD_3OD, 1 mM).

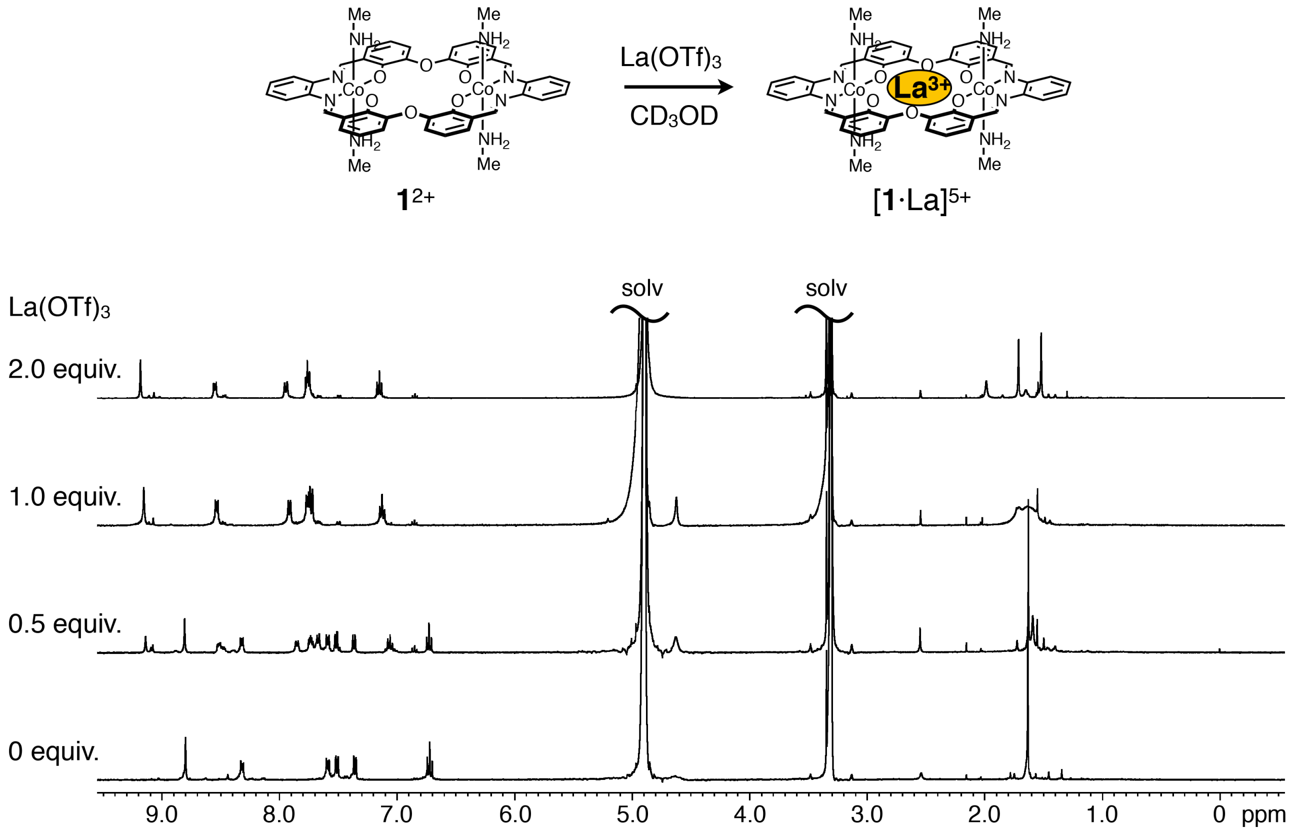

Supplementary Figure 10. ^{1}H NMR spectral changes of **1**(OTf)$_2$ upon the addition of La(OTf)$_3$ (400 MHz, CD$_3$OD, 1 mM, 120 h after mixing).

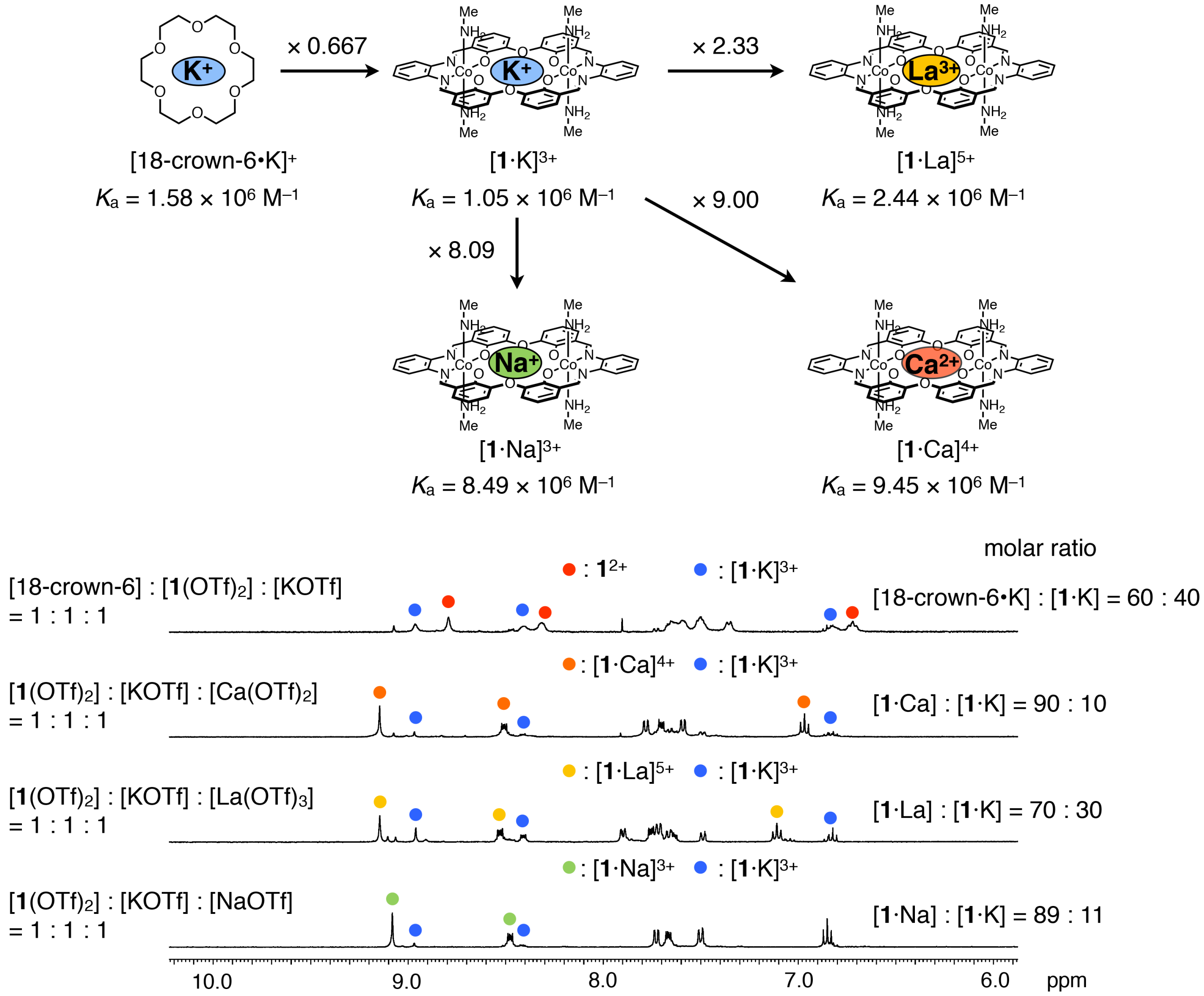

Supplementary Figure 11. ^{1}H NMR spectra for competitive experiments (400 MHz, CD_3OD, [**1**$(OTf)_2$] = 1 mM).

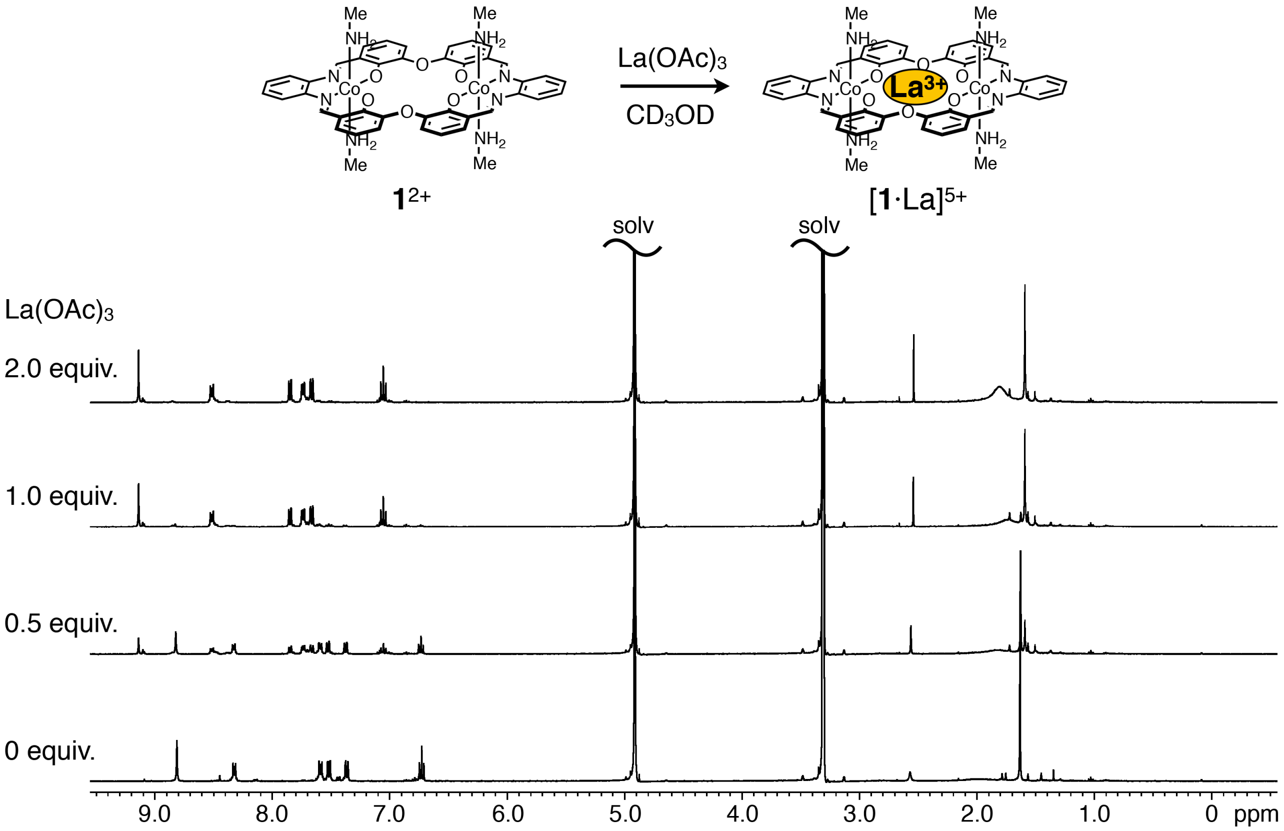

Supplementary Figure 12. ^{1}H NMR spectral changes of **1**(OTf)$_2$ upon the addition of La(OAc)$_3$ (400 MHz, CD$_3$OD, 1 mM, 1 h after mixing).

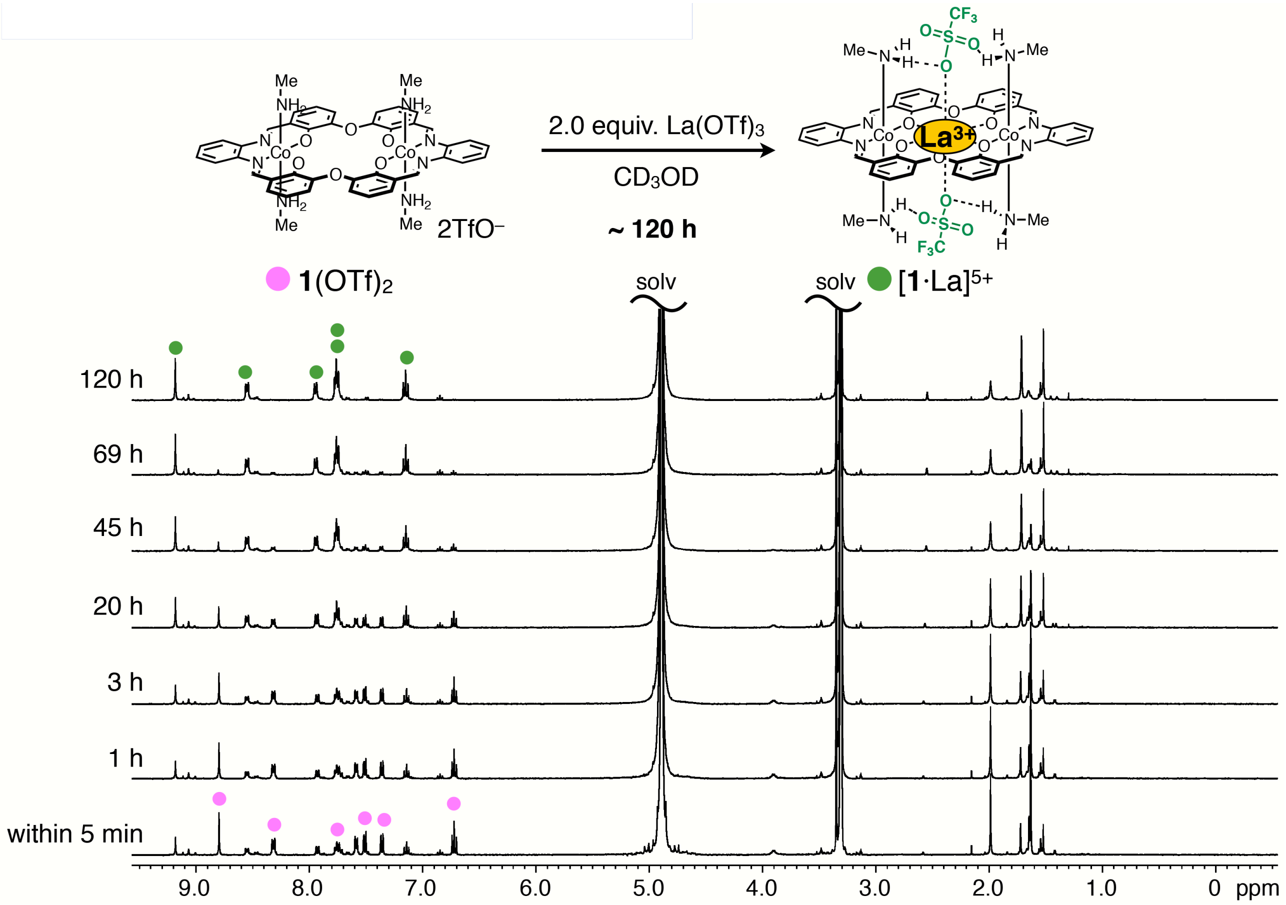

Supplementary Figure 13. Time-dependent ^{1}H NMR spectra of **1**(OTf)$_2$ upon the addition of 2.0 equiv. of La(OTf)$_3$ (400 MHz, CD_3OD, 1 mM).

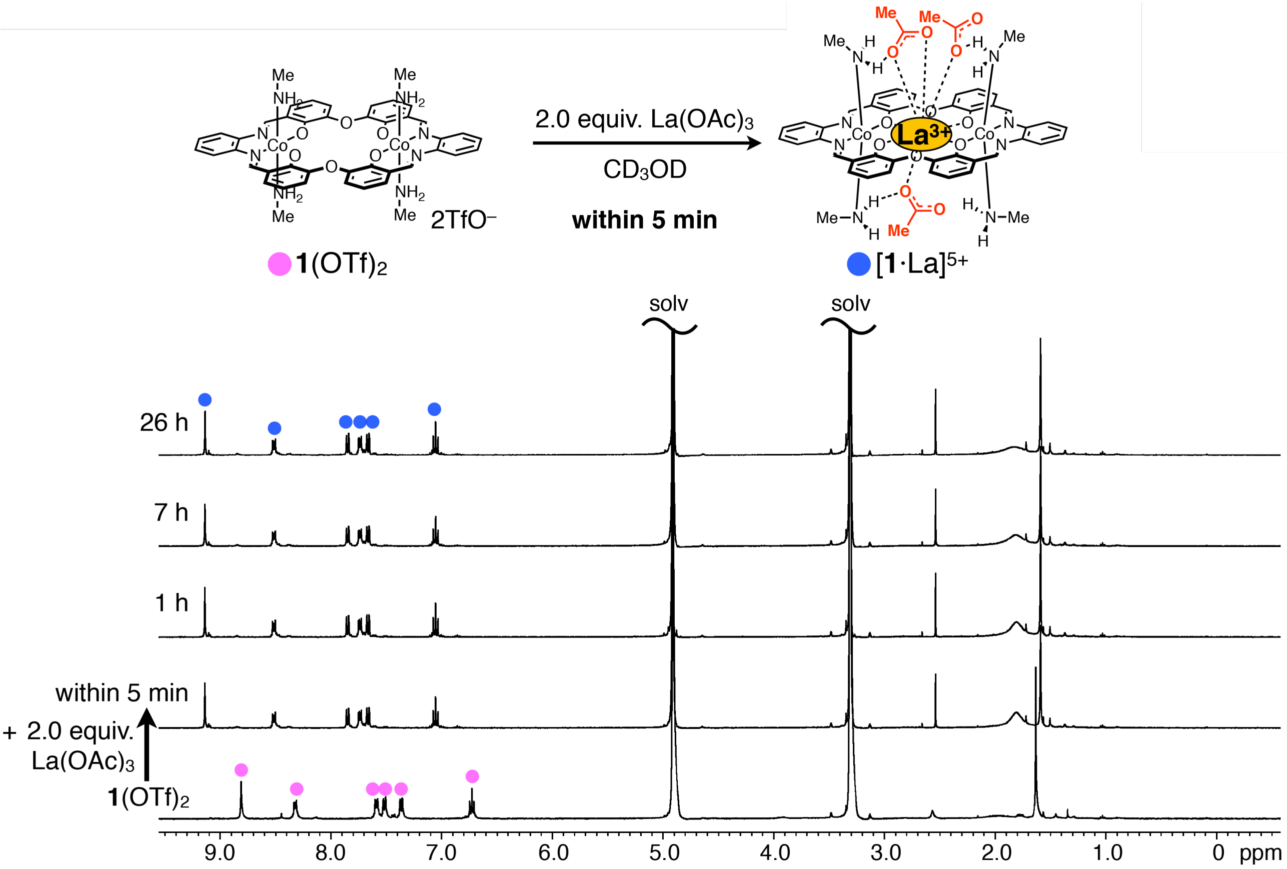

Supplementary Figure 14. Time-dependent ^{1}H NMR spectra of **1**(OTf)$_2$ upon the addition of 2.0 equiv. of La(OAc)$_3$ (400 MHz, CD$_3$OD, 1 mM).

Supplementary Table 1. Crystallographic data for **1**(OTf)$_2$, and [**1**•Na(OTf)$_2$](OTf).

	**1**(OTf)$_2$•(CH$_3$CN)$_4$(H$_2$O)	[**1**•Na(OTf)$_2$](OTf)•(Et$_2$O)(MeOH)$_3$(H$_2$O)
Formula	$C_{54}H_{58}Co_2F_6N_{12}O_{13}S_2$	$C_{54}H_{68}Co_2F_9N_8NaO_{20}S_3$
Formula weight	1379.10	1557.19
Temperature (K)	83	93
Crystal size (mm^3)	0.40 × 0.10 × 0.10	0.20 × 0.10 × 0.05
Crystal system	monoclinic	monoclinic
Space group	$P2_1/c$	$P2_1/c$
a (Å)	9.340(2)	10.5611(15)
b (Å)	21.207(5)	18.785(2)
c (Å)	14.903(3)	17.438(3)
α (deg)	90	90
β (deg)	98.389(6)	96.893(4)
γ (deg)	90	90
V (Å^3)	2920.3(11)	3434.4(8)
Z	2	2
D_{calcd} (g cm^{-3})	1.568	1.506
Collected reflections	29569	34966
Unique reflections	6628	6728
R_{int}	0.0544	0.0642
$2\theta_{max}$	55.01	51.998
F_{000}	1420	1604
μ(MoKα)	0.733	0.679
Limiting indices	$-11 \leq h \leq 12$	$-11 \leq h \leq 13$
	$-27 \leq k \leq 27$	$-23 \leq k \leq 20$
	$-17 \leq l \leq 19$	$-21 \leq l \leq 21$
Restraints/parameters	2/436	85/581
Goodness of fit (F^2)	1.072	1.124
$R1$ ($I > 2\sigma(I)$)	0.0566	0.0703
$wR2$ ($I > 2\sigma(I)$)	0.1511	0.1771
$R1$ (all data)	0.0644	0.0872
$wR2$ (all data)	0.1618	0.1952

Supplementary Table 2. Crystallographic data for [**1**•K(OTf)$_2$](OTf), and [**1**•Ca(OTf)$_2$](OTf)$_2$.

	[**1**•K(OTf)$_2$](OTf)•(MeOH)$_{1.8}$	[**1**•Ca(OTf)$_2$](OTf)$_2$.
Formula	$C_{48.80}H_{51.20}Co_2F_9KN_8O_{16.80}S_3$	$C_{48}H_{44}CaCo_2F_{12}N_8O_{18}S_4$
Formula weight	1442.71	1535.09
Temperature (K)	93	90
Crystal size (mm^3)	0.26 × 0.06 × 0.03	0.10 × 0.06 × 0.05
Crystal system	triclinic	orthorhombic
Space group	*P*1	*Ccca*
a (Å)	9.7776(6)	13.126(4)
b (Å)	10.5777(7)	17.452(7)
c (Å)	15.5321(10)	24.042(4)
α (deg)	104.847(4)	90
β (deg)	105.118(4)	90
γ (deg)	100.507(4)	90
V (Å^3)	1444.59(17)	5507(3)
Z	1	4
D_{calcd} (g cm^{-3})	1.658	1.851
Collected reflections	11212	21890
Unique reflections	7290	2341
R_{int}	0.0447	0.1238
$2\theta_{max}$	140.14	50.00
F_{000}	736	3112
μ(CuKα)	7.096	–
μ(MoKα)	–	0.972
Limiting indices	$-11 \leq h \leq 10$	$-15 \leq h \leq 13$
	$-12 \leq k \leq 12$	$-20 \leq k \leq 17$
	$-18 \leq l \leq 18$	$-28 \leq l \leq 28$
Restraints/parameters	1312/1005	280/384
Goodness of fit (F^2)	1.039	1.220
$R1$ ($I > 2\sigma(I)$)	0.0853	0.1010
$wR2$ ($I > 2\sigma(I)$)	0.2031	0.2339
$R1$ (all data)	0.1033	0.1418
$wR2$ (all data)	0.2185	0.2602

Supplementary Table 3. Crystallographic data for [**1**•La(OAc)$_3$](OTf)$_2$.

	[**1**•La(OAc)$_3$](OTf)$_2$•(H$_2$O)(MeOH)
Formula	$C_{53}H_{59}Co_2F_6N_8O_{20}S_2$
Formula weight	1562.97
Temperature (K)	93
Crystal size (mm^3)	0.06 × 0.04 × 0.01
Crystal system	triclinic
Space group	*P*–1
a (Å)	10.436(3)
b (Å)	14.875(4)
c (Å)	20.875(5)
α (deg)	75.352(7)
β (deg)	80.873(7)
γ (deg)	87.247(8)
V (Å^3)	3095.5(14)
Z	2
D_{calcd} (g cm^{-3})	1.677
Collected reflections	20987
Unique reflections	10842
R_{int}	0.1779
$2\theta_{max}$	50.594
F_{000}	1580
μ(MoKα)	1.373
Limiting indices	$-12 \leq h \leq 8$
	$-17 \leq k \leq 17$
	$-24 \leq l \leq 25$
Restraints/parameters	891/941
Goodness of fit (F^2)	1.038
$R1$ ($I > 2\sigma(I)$)	0.1098
$wR2$ ($I > 2\sigma(I)$)	0.2553
$R1$ (all data)	0.2241
$wR2$ (all data)	0.3141

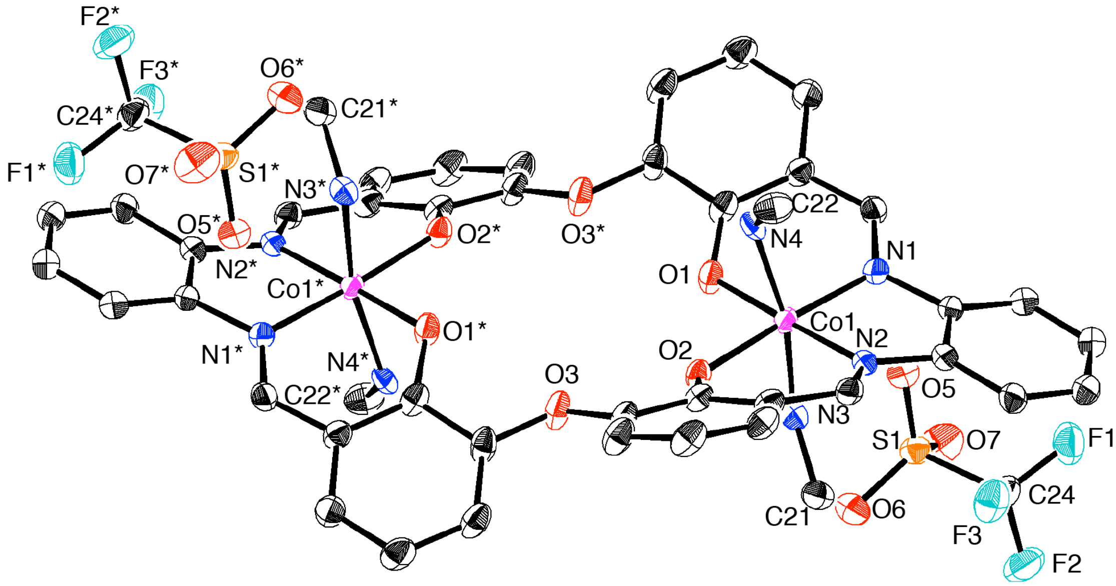

Supplementary Figure 15. X-ray crystal structure of **1**$(OTf)_2$ with thermal ellipsoids plotted at the 50% probability level. Hydrogen atoms, solvent molecules, and minor components disordered atoms are omitted for clarity.

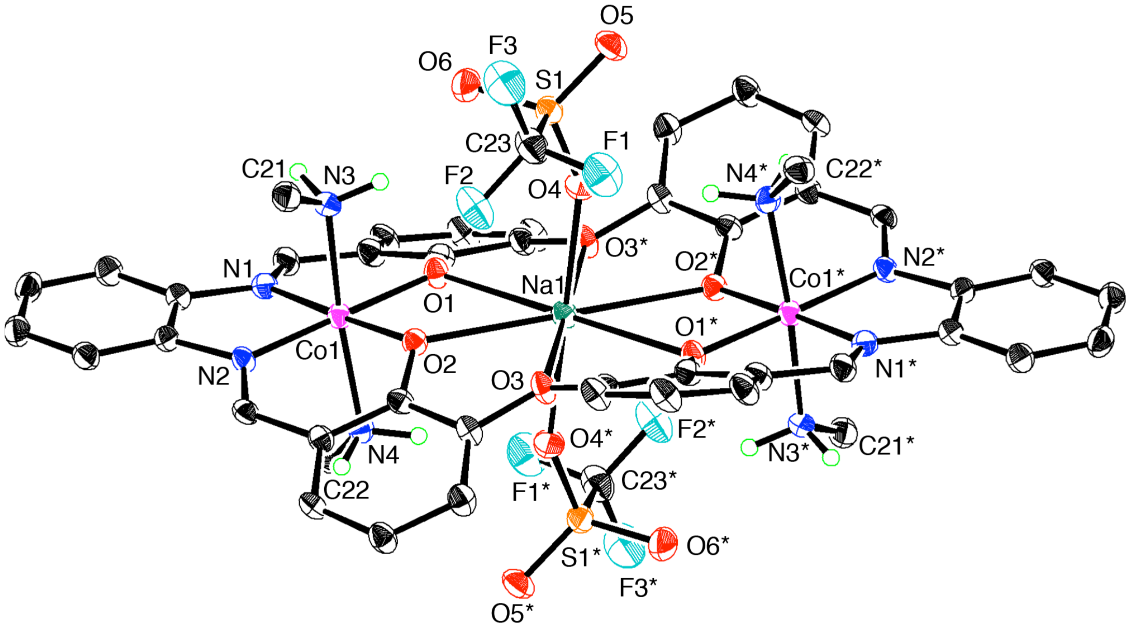

Supplementary Figure 16. X-ray crystal structure of [**1**•$Na(OTf)_2$](OTf) with thermal ellipsoids plotted at the 30% probability level. Hydrogen atoms are omitted for clarity except for those of the NH_2 groups. The solvent molecules and one triflate anion are also omitted.

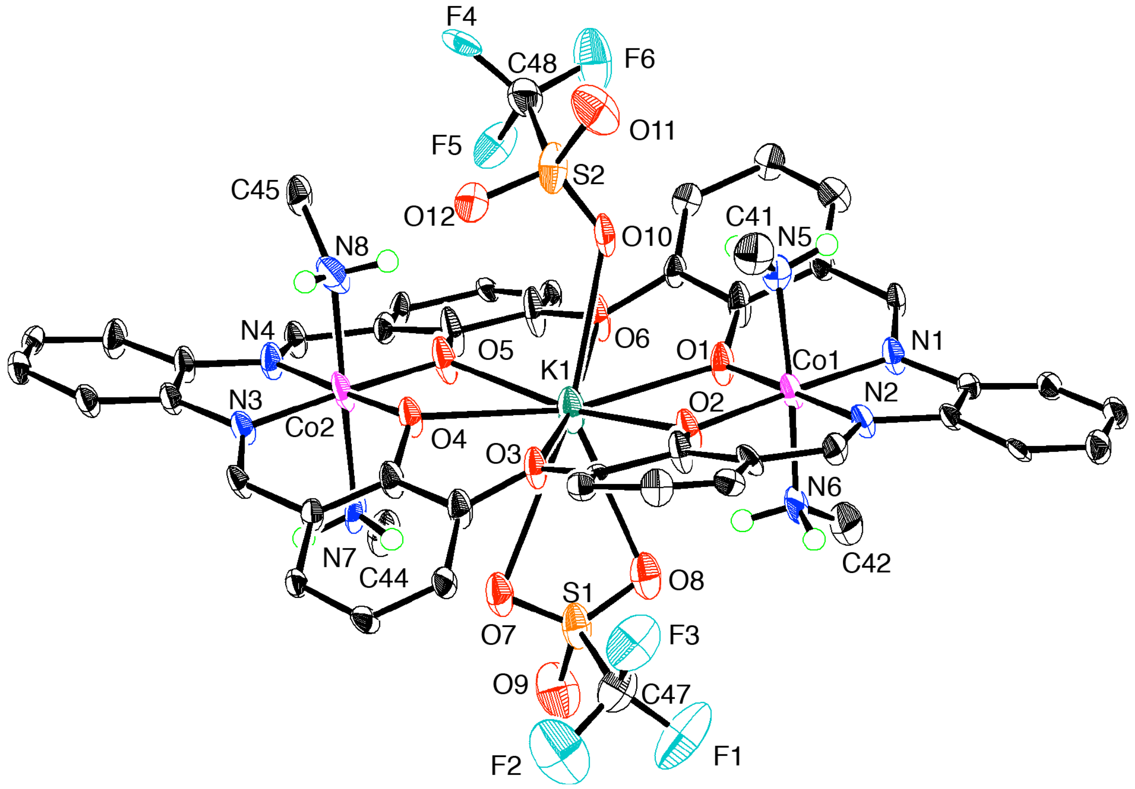

Supplementary Figure 17. X-ray crystal structure of [**1**•K(OTf)$_2$](OTf) with thermal ellipsoids plotted at the 50% probability level. Hydrogen atoms are omitted for clarity except for those of NH_2 groups. The solvent molecules, one of the triflate anions, and minor components disordered atoms are also omitted.

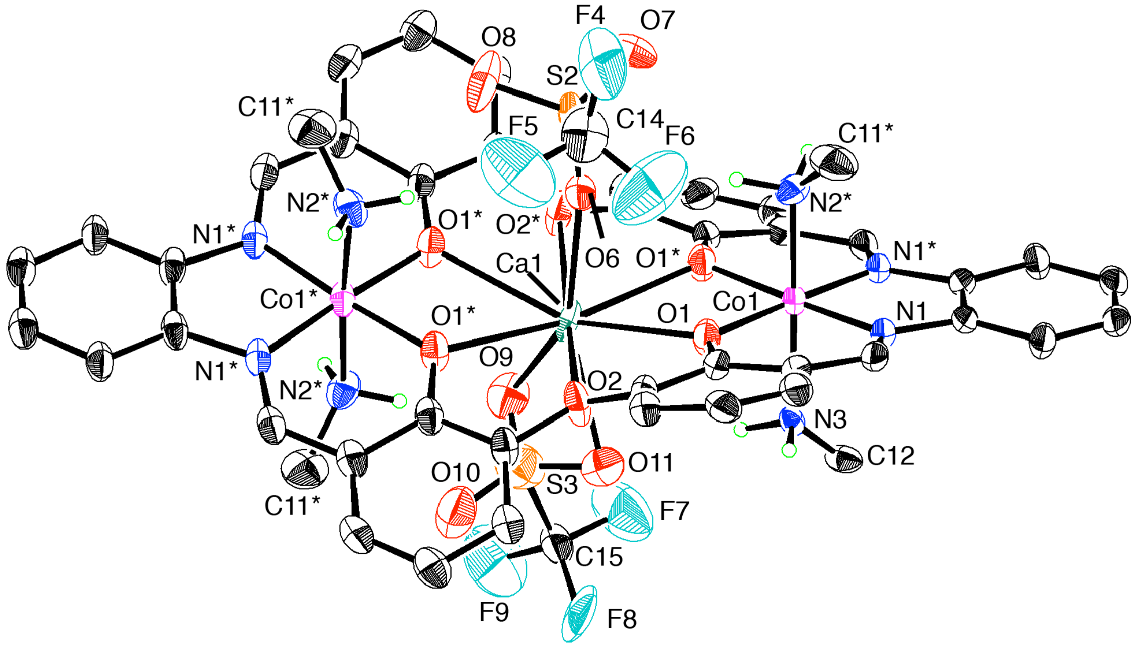

Supplementary Figure 18. X-ray crystal structure of [**1**•Ca(OTf)$_2$](OTf)$_2$ with thermal ellipsoids plotted at the 20% probability level. Hydrogen atoms are omitted for clarity except for the NH_2 groups. The solvent molecules, two triflate anions, and minor components disordered atoms are also omitted.

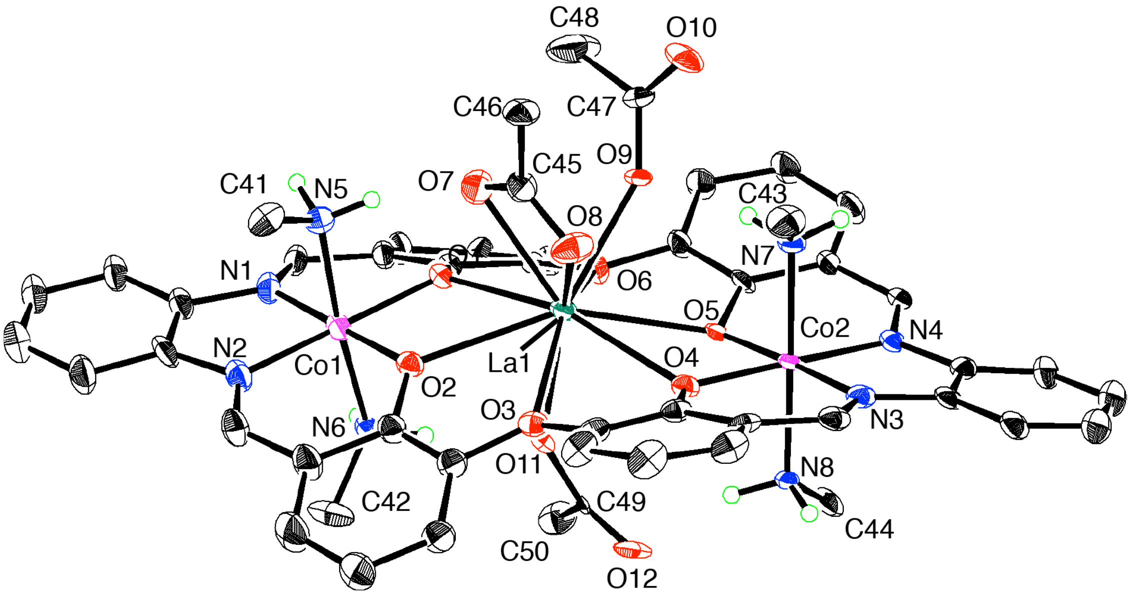

Supplementary Figure 19. X-ray crystal structure of [**1**•La(OAc)$_3$](OTf)$_2$ with thermal ellipsoids plotted at the 30% probability level. Hydrogen atoms are omitted for clarity except for those of the NH_2 groups. The solvent molecules and two triflate anions are also omitted.

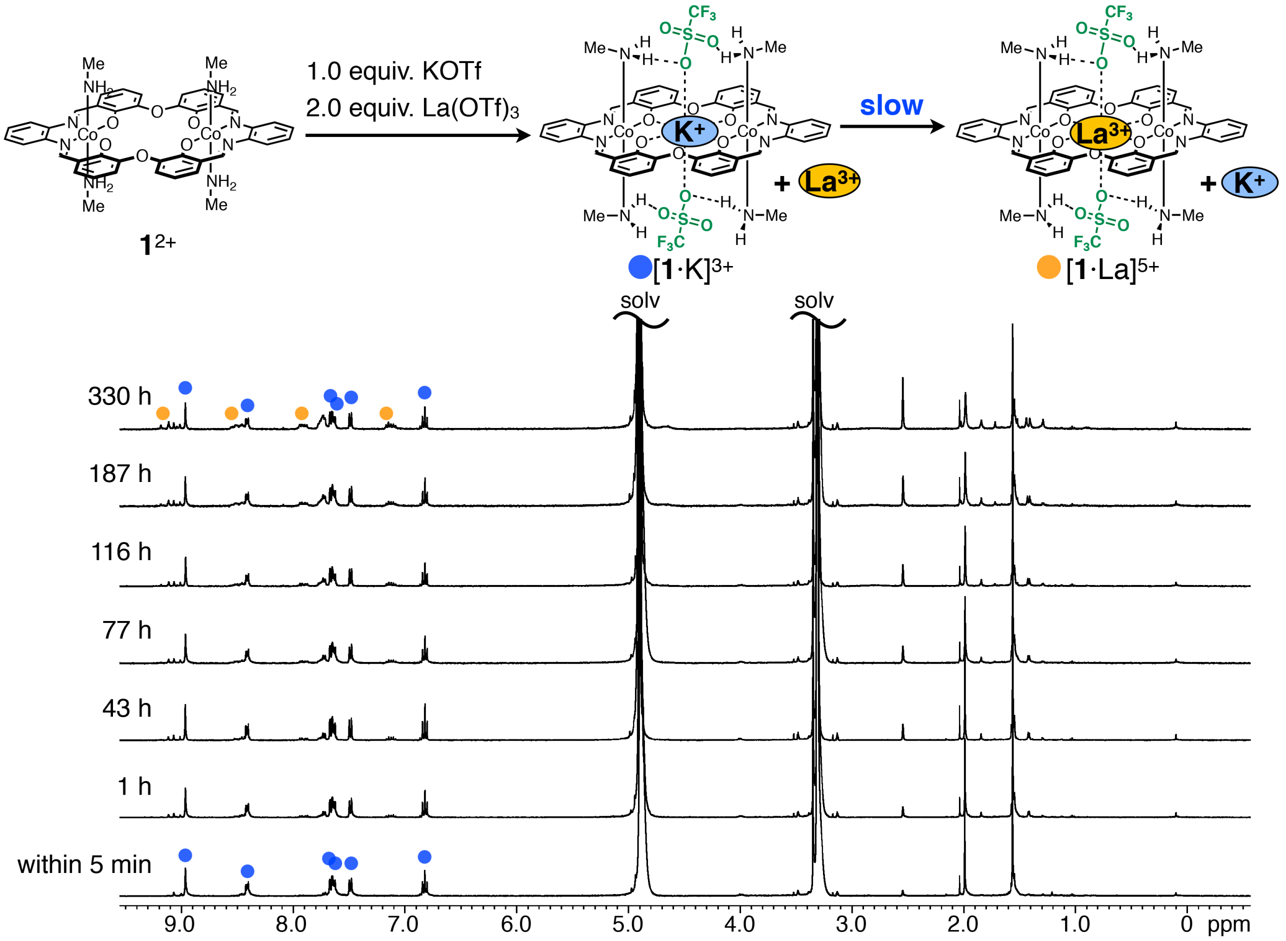

Supplementary Figure 20. Time-dependent ^{1}H NMR spectra of **1**(OTf)$_2$ upon the addition of 2.0 equiv. of La(OTf)$_3$ and 1.0 equiv. of KOTf (400 MHz, CD$_3$OD, 1 mM).

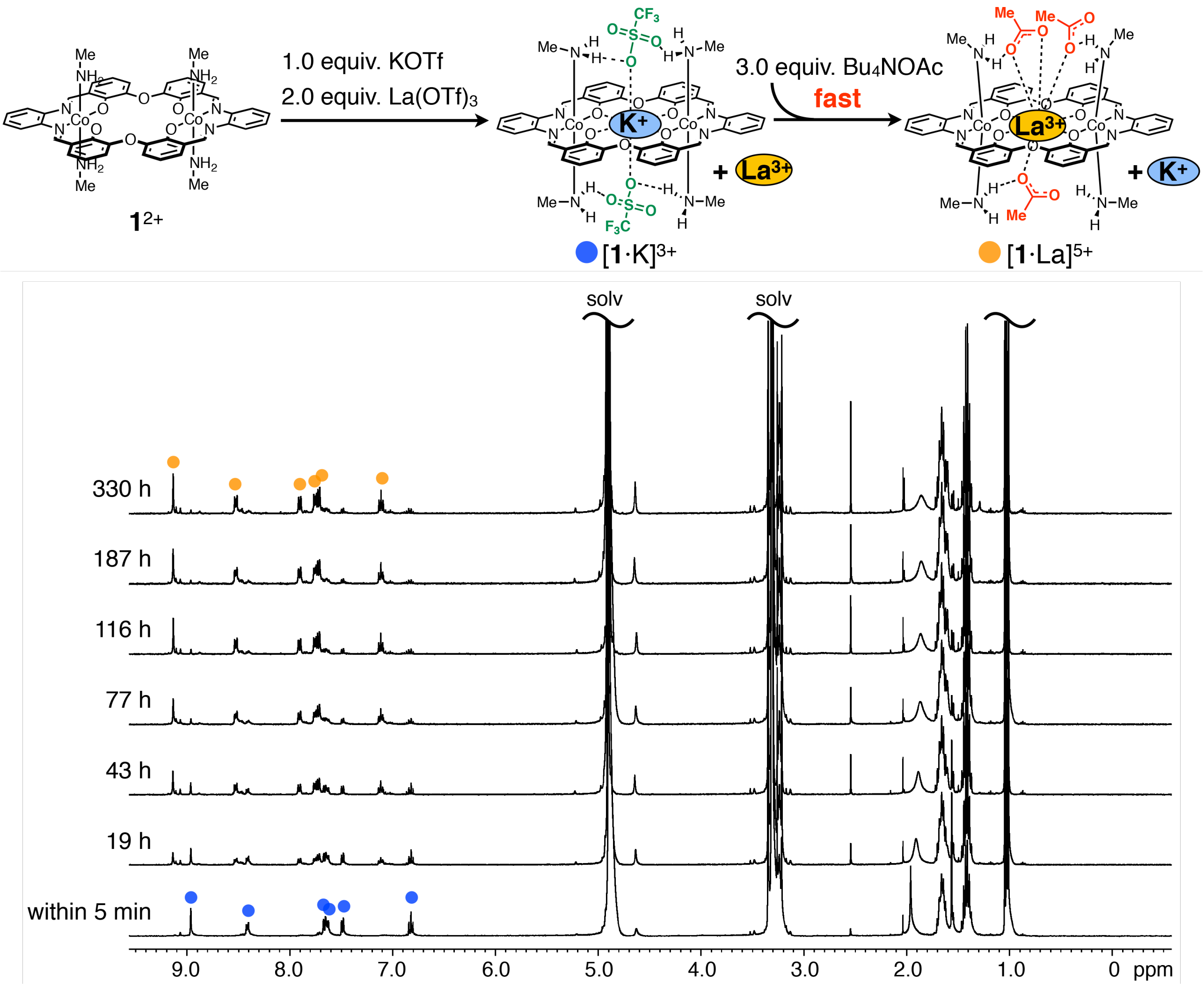

Supplementary Figure 21. Time-dependent ^{1}H NMR spectra of **1**$(OTf)_2$ upon the addition of 2.0 equiv. of $La(OAc)_3$, 1.0 equiv. of KOTf, and 3.0 equiv. of Bu_4NOAc (400 MHz, CD_3OD, 1 mM).

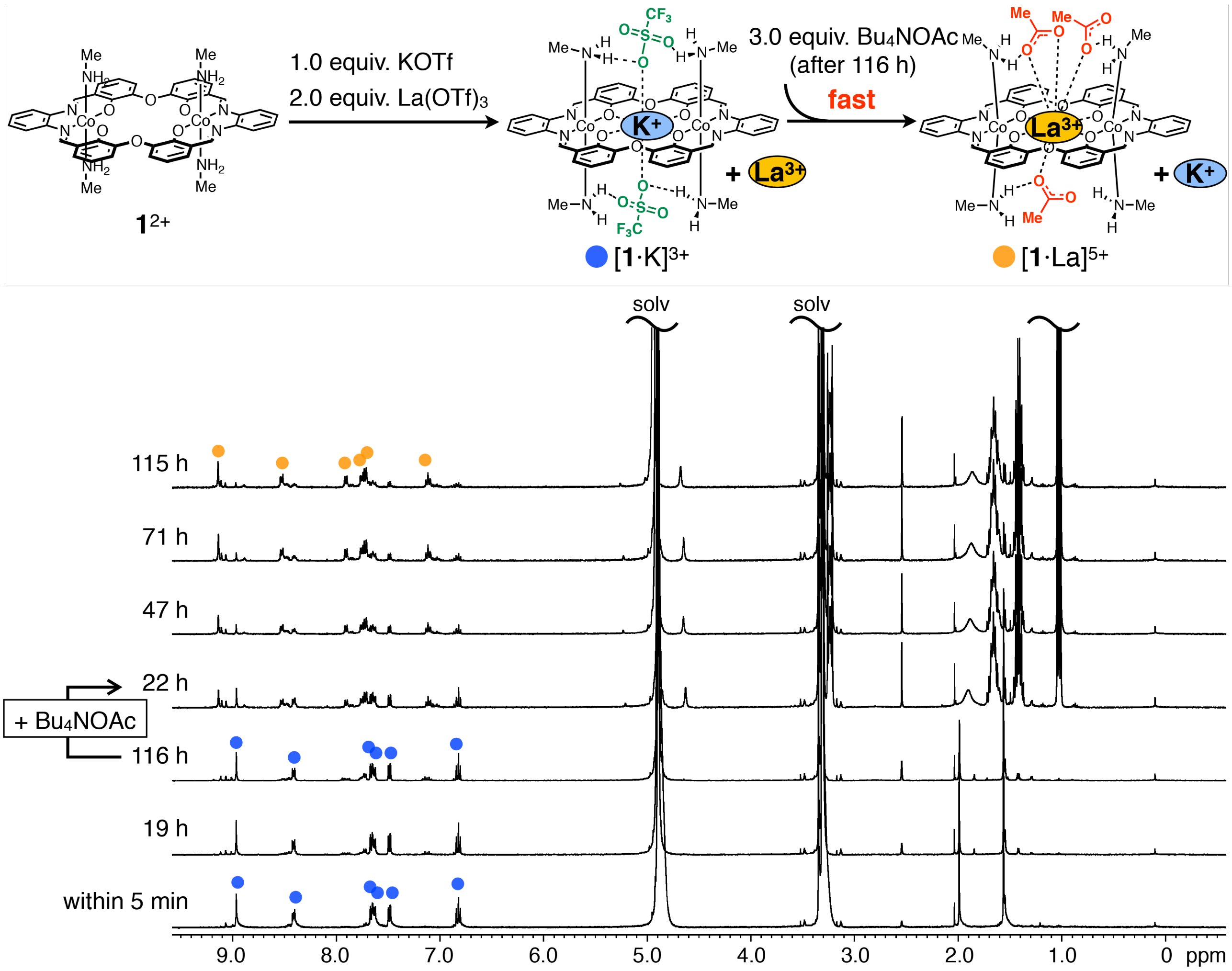

Supplementary Figure 22. Time-dependent ^{1}H NMR spectra of **1**$(OTf)_2$ upon the addition of 2.0 equiv. of $La(OAc)_3$, 1.0 equiv. of KOTf. 3.0 equiv. of Bu_4NOAc was added after 116 h after complexation (400 MHz, CD_3OD, 1 mM).

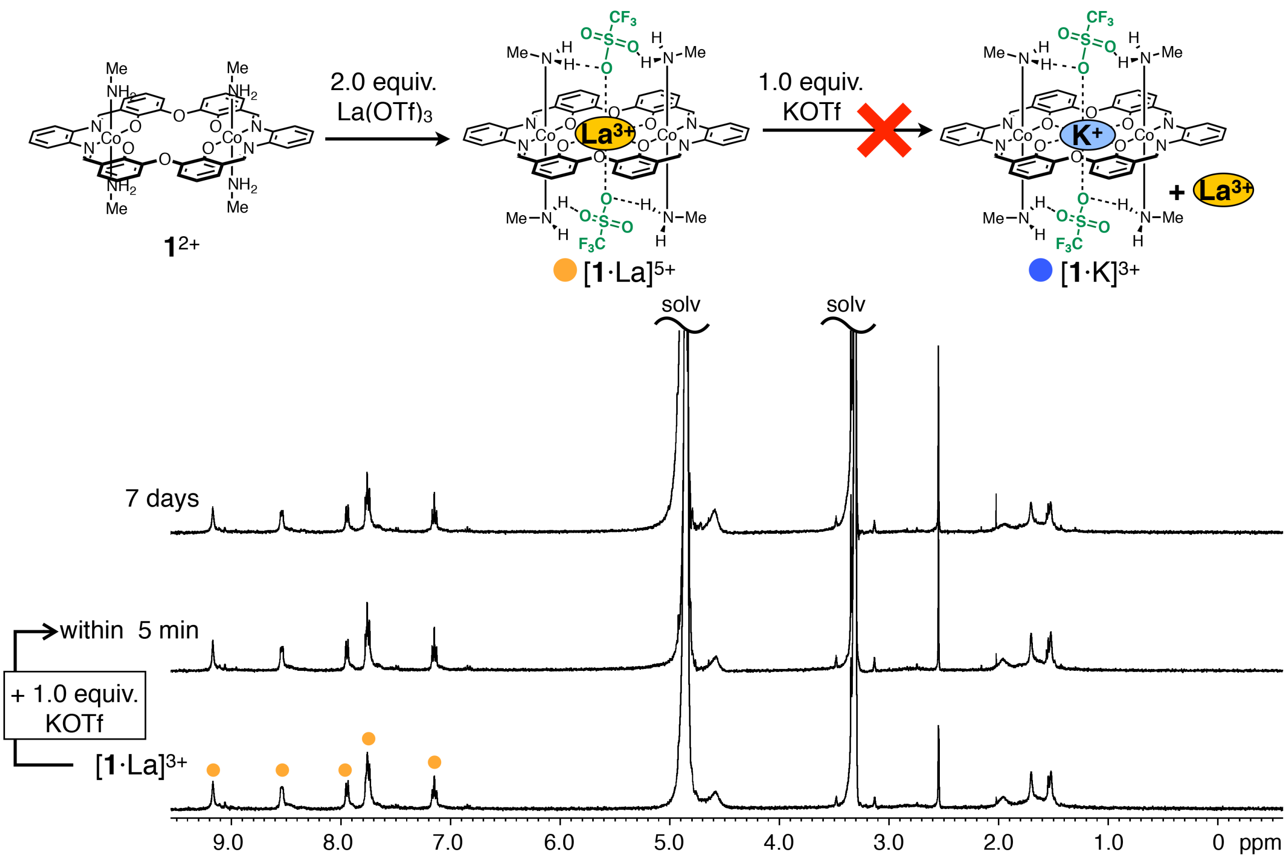

Supplementary Figure 23. ^{1}H NMR spectral changes of [**1**•La(OTf)$_2$](OTf)$_3$ upon the addition of 1.0 equiv. of KOTf (400 MHz, CD_3OD, 1 mM).
